# Clinoptilolite Microparticles as Carriers of Catechin-Rich *Acacia catechu* Extracts: Microencapsulation and In Vitro Release Study

**DOI:** 10.3390/molecules26061655

**Published:** 2021-03-16

**Authors:** Zvezdelina Yaneva, Donika Ivanova, Nikolay Popov

**Affiliations:** 1 Chemistry Unit, Department of Pharmacology, Animal Physiology and Physiological Chemistry, Faculty of Veterinary Medicine, Students Campus, Trakia University, 6000 Stara Zagora, Bulgaria; donika_georgiewa@abv.bg; 2 Mineralagro-Z Ltd., 1000 Sofia, Bulgaria; popov_n_1944@abv.bg

**Keywords:** *Acacia catechu*, catechin, Clinosorbent-5, encapsulation, in vitro release

## Abstract

The main goal of the present study was to investigate the microencapsulation, in vitro release capacity and efficiency of catechin-rich *Acacia catechu* extract by Clinosorbent-5 (CLS-5) microparticles by in-depth detailed analyses and mathematical modelling of the encapsulation and in vitro release kinetics behaviour of the polyphenol-mineral composite system. The bioflavanol encapsulation and release efficiency on/from the mineral matrix were assessed by sorption experiments and interpretative modelling of the experimental data. The surface and spectral characteristics of the natural bioactive substance and the inorganic microcarrier were determined by Fourier Transform Infrared Spectroscopy (FTIR) and Ultraviolet/Visible (UV/Vis) spectrophotometric analyses. The maximum extent of catechin microencapsulation in acidic medium was 32%. The in vitro release kinetics study in simulated enzyme-free gastric medium (pH = 1.2) approved 88% maximum release efficiency achieved after 24 h. The in vitro release profile displayed that the developed bioflavanol/clinoptilolite microcarrier system provided sustained catechin in vitro release behaviour without an initial burst effect. Thus, the results from the present study are essential for the design and development of innovative catechin-CLS-5 microcarrier systems for application in human and veterinary medicine.

## 1. Introduction

The potential applications of clinoptilolite as a promising drug-carrier material for biomedicine and pharmaceutics were intensively studied and reviewed, especially up to the first decade of the XXI century. Later on, a significant decline of such type of investigations was observed, probably due to the emerging interest in various biopolymer formulations as efficient drug excipients. However, nowadays, a revival of studies on the potential of the natural zeolitic tuff as a drug-carrier candidate is of utmost importance due to its essential and indisputable physicochemical properties—non-toxicity, thermal stability, expanded surface area, exceptional ion-exchange and adsorption capacity towards ions and organic molecules [1], biocatalytic potential and a number of biological activities [2].

Clinoptilolites displayed antioxidative and anticancer activity [3,4] and support the immune activity [5]. They are stable in the gastrointestinal tract, and due to their unique selective sorption potential, they could adsorb toxins, heavy metals and free radicals from the body and excretes [6,7,8].

Topashka-Ancheva et al. established that the structure of chromosomes and red blood cells, as well as the mitotic index and erythropoiesis, was significantly improved by the supplementation of Pb-exposed laboratory mice the with clinoptilolite sorbent clinosorbent KLS-10-MA, based on natural Bulgarian clinoptilolite. These findings ascertained that the clinoptilolite sorbent KLS-10-MA exerted a significant favourable effect and could be applied as a reliable tool for detoxification of human and animal organisms chronically poisoned by heavy metals [9].

Besides, considering the close relation of biochemical processes to ion exchange, adsorption and catalysis, natural and/or modified clinoptilolites could offer a significant contribution to the pharmaceutical industry and medicine in the near future [10].

In this respect, the possible encapsulation of ions and drug molecules in the alumosilicate open framework, and their subsequent controlled release, are among the most valuable pharmacological applications of zeolite type materials [11,12,13,14].

The novel study of Strzempek et al. displayed the potential of a zeolite, composed of zeolite ferrierite layers with embedded choline cations, as an efficient ciprofloxacin and piracetam carrier. The established differences in the release behaviour of both drugs were attributed to strong host–guest interactions responsible for the modified, slowed down release of piracetam, while the better solubility and higher release rate of ciprofloxacin was due to protonation and subsequent weaker interactions between the drug molecules and zeolite [15].

Earlier studies reported the potential of clinoptilolite as a carrier of conventional pharmaceuticals, such as nonsteroidal anti-inflammatory drugs (ketoprofen, diclofenac), antibiotics, salicylic acid, metformin HCl, etc. [13,16]. Jevtic et al. established a two-stage in vitro release mechanism of salicylate ions from benzalkonium chloride-modified clinoptilolite with 50% release efficiency within the first 15 min, followed by 30% slow release within the next 5 h [13]. A study on the adsorption of the anti-tuberculosis drug isoniazid in mordenite, faujasite-Y and beta zeolite indicated that total drug sorption was due to partial internal surface adsorption in the zeolite channels and partial absorption in the external surface, which is directly related to the ratio total superficial area/quantity of crystalline isoniazid in the mineral frameworks [17]. Mavrodinova et al. reported a successful simplified procedure for the preparation of a dual Ag-sulfadiazine drug delivery system based on zeolite Y-carrier. It was established that the solid-state sulfadiazine deposition method provoked restriction of the reaction between the drug and silver within the limits of inter-atomic interaction. The latter resulted in total but prolonged drug release, which outlined the perspective of the designed drug release system for burn injury topical applications [18].

Scientific literature presents a great number of studies on the bioactivity and major health benefits of various naturally derived polyphenols and bioflavonoids [19]. The major concerns of their direct administration as therapeutic agents arise from limitations associated with low bioavailability, poor solubility in the gastrointestinal fluids, sensitivity to oxidation, incomplete absorption from the gastrointestinal tract, reduced or no biological activity and toxicity. To cope with these restrictions, modern scientific studies have been directed towards the design of innovative carrier systems. However, limited number of investigations is subjected to the potential of natural mineral materials as carriers of natural biologically active compounds.

Zeolite Y-gelatin composite was used as a drug-carrier matrix for oral drug delivery of the monocyclic sesquiterpene zerumbone, isolated from *Zingiber Zerumbet (L) Smith* rhizomes [20]. Karimi et al. reported 37.76%–61% encapsulation efficiency of pH-sensitive zeolite-based nanocomposites towards the hydrophobic curcumin [21]. Silica-based and microporous zeolite materials were used as carriers for the preparation, by solid-state dry mixing method, of resveratrol-loaded delivery systems. The applied method proved to be effective for the encapsulation, sustained in vitro release (100% in simulated gastric and intestinal fluid after 5 h), stabilisation of the drug and enhanced solubility [22].

In addition to effective encapsulation and sustained release, a number of studies reported increased synergistic bioactivity of biologically active substances-loaded zeolite formulations. Tomeckova et al. established that clinoptilolites modified with the pharmacologically active natural substances escin and horse chestnut extract displayed increased anticancer activity in adenocarcinomic human alveolar basal epithelial cells [23]. A zeolite/salicylic acid composite displayed significant antibacterial activity against *Escherichia coli* (*E. coli*) and *Staphylococcus aureus* (*S. aureus*) in a dose of 0.5 g/100 cm^3^ within 1 h of contact [13]. Two independent tests on L929 mice fibroblasts showed that two-dimensional (2D) zeolite with ferrierite layer topology may be safely used as a carrier of drug molecules in moderate concentrations without negative effect on the cells’ viability or proliferation rate [15]. The application of clinoptilolite as a gastric antacid, antidiarrheic agent and a potential adjuvant in anticancer therapy has also been reported [1,21,24,25].

Our previous investigations on the host–guest interactions, the mechanism of caffeine and d,l-α-tocopherol acetate encapsulation and in vitro release demonstrated the applicability and efficiency of natural and modified Bulgarian zeolites as drug-carriers [26,27].

The lack of studies on the potential and efficiency of natural Bulgarian clinoptilolites as excipients of bioflavonoids, especially catechins, provoked the present investigations. The objectives of the present study were determined by the fact that detailed knowledge on the mass transfer mechanism of drug release is among the major prerequisites to the systematic improvement of the design and activity of drug delivery systems [28].

Thus, the main goal was to investigate the microencapsulation and in vitro release capacity and efficiency of catechin-rich *Acacia catechu* extract by Clinosorbent-5 (CLS-5) microparticles by in-depth detailed analyses and mathematical modelling of the encapsulation and in vitro release kinetics behaviour of the polyphenol-mineral composite system.

## 2. Results and Discussion

### 2.1. CLS-5 Characterisation

The chemical content and physicochemical properties of CLS-5 are presented in Reference [29]. The comparative estimation of the data for two types of natural clinoptilolites (from Beli Plast and Beli Bair deposits, Bulgaria) and CLS-5 indicated that the modified mineral was characterised with the highest CaO and Na_2_O content, 1.5 times higher total cation exchange capacity and 1.1–1.5 times higher sorption capacity towards Sr^+2^, Cs^+^, Pb^+2^ and Cd^+2^, as well as alkaline pH. The ratio SiO_2_/Al_2_O_3_ of zeolitic tuffs has been determined as a significant indicator, which is directly proportional to their acid stability [9]. The relatively high SiO_2_/Al_2_O_3_ ratio of CLS-5 (SiO_2_/Al_2_O_3_ = 6.13) is indicative of its stability in simulated enzyme-free gastric fluid.

**Figure 1:**
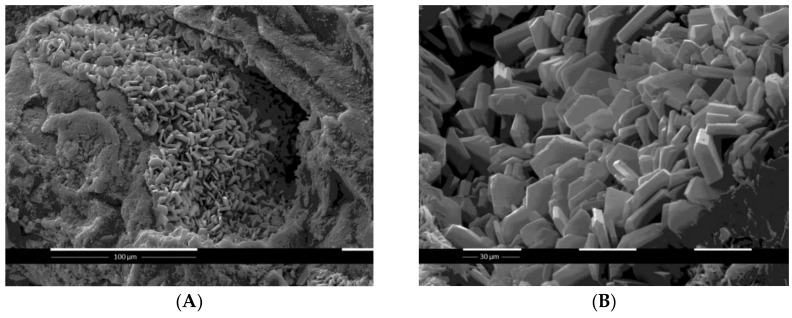
SEM images of druse of recrystallised clinoptilolite at magnification: (**A**) ×1000 and (**B**) ×2000.

The high-resolution Scanning Electron Microscopy SEM images of recrystallised clinoptilolite druses at magnification ×1000 (Figure 1A) and ×2000 (Figure 1B) show the well-developed porous structure of the mineral microparticles. Obviously, most of the crystals are non-agglomerated and well-defined as plates/slats with prismatic form and regular morphology due to the high concentration of silica. The absence of fibrous phases represents the high phase purity of CLS-5.

The latter observations are a prerequisite for the suitability of CLS-5 for application as a medical device and/or dietary supplement and justify its potential as a drug-carrier candidate for therapeutic purposes.

With regard to the production of CLS-5, the documentation was collected and approved by the Department of Health Prevention and State Sanitary Control at the Ministry of Health, Bulgaria (Letter No. 33-00-183/1993).

### 2.2. UV-Vis Spectrophotometric Analyses

The concentration of catechin in the spray-dried extract of *Acacia catechu* was determined by a non-derivative (ND) and first derivative (FD) UV-Vis spectrophotometric methodology developed by us at pH 7.9 and pH 4.0 [30]. The method is characterised with significant average percent recovery (≈97%) of the bioflavonoids in the raw plant extracts. Due to the fact that catechin is unstable and easily oxidised, especially in alkaline medium, all UV-Vis analyses and encapsulation experiments were conducted at pH = 4. The ethanol extract solutions were acidified with 2N CH_3_COOH. The choice of acetic acid was dictated by the fact that the newly designed catechin-clinoptilolite complexes are intended for biomedical applications.

*Acacia catechu* extracts with initial catechin concentrations in the range *C*_0_ = 50–200 µg/cm^3^ were used in the encapsulation experiments.

**Figure 2:**
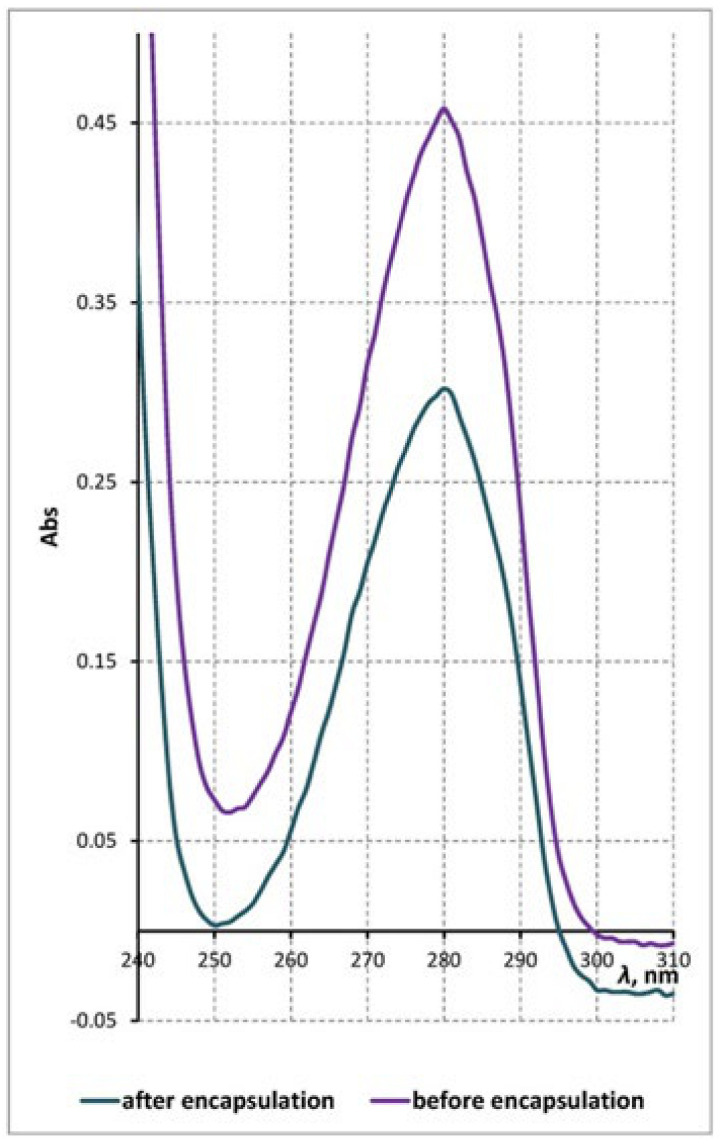
UV-Vis spectra of *Acacia catechu* extract before (*C*_0_ = 50 μg/cm^3^) and after encapsulation (*C_e_* = 32.7 μg/cm^3^).

Figure 2 shows the UV absorbance spectra of *Acacia catechu* ethanol extract before and after the contact with the clinoptilolite microparticles. The analysis of the spectra indicates that the contact of the bioorganic molecules with the carrier framework does not produce any damage on the structure of the organic molecules.

### 2.3. FTIR Analyses

**Figure 3:**
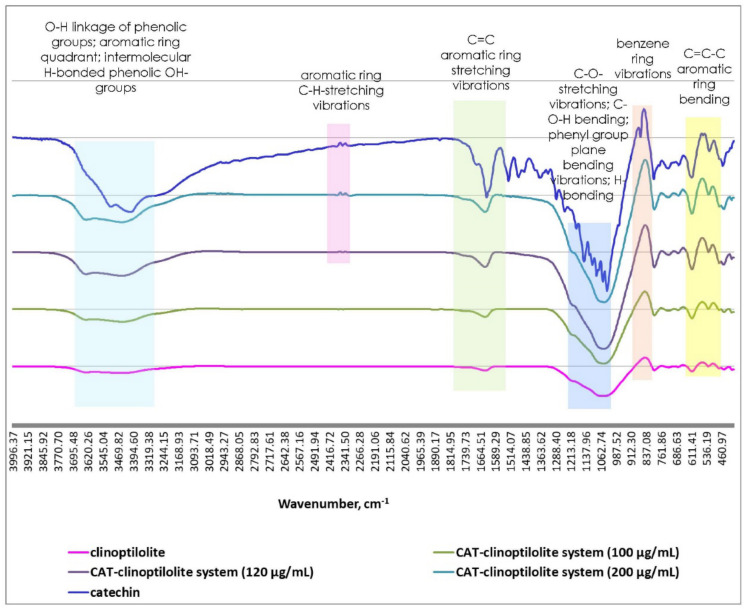
FTIR spectra of spray-dried catechin powder, clinoptilolite and catechin-loaded clinoptilolite.

The FTIR spectra of spray-dried catechin powder, clinoptilolite and three catechin-clinoptilolite complexes (with initial bioflavanol concentrations *C*_0_ = 100, 120 and 200 µg/cm^3^) are displayed in Figure 3. The wavelengths of the characteristic bands and the corresponding designations are presented in Supplementary Materials Table S1.

The absorption bands at 3516 and 1282–1029 cm^−1^ in the catechin spectrum are related to hydrogen bond formation. The slight shift of these wavenumbers to lower frequencies (3438 and 1060 cm^−1^, respectively), which were observed in the spectra of catechin-clinoptilolite microparticles, depicts formation of H-bonds between catechin -OH-groups and the two types of CLS-5 OH-groups: lattice termination silanol groups, located on the external surface, and bridging OH-groups with Bronsted acidity. Furthermore, the spitted peaks at 879 and 844 cm^−1^ are indicative of the presence of a benzene ring in the polyphenol molecule. This band was found to shift to a lower frequency (835 cm^−1^), to broaden and to become sharper with decreasing catechin concentration in the three studied catechin-CLS-5 samples. These observations undoubtedly confirm the encapsulation of catechin in the mineral microparticles. Similar trends were observed for the deviations of the 565 and 503 cm^−1^ spectra that are responsible for C=C–C-aromatic ring asymmetric bending and C–H out-of-plane aromatic ring bending. The gradual decrease of the peak intensities was directly proportional to the decrease of catechin initial concentration in the polyphenol-mineral composite systems [27,31,32,33,34].

The comparative analyses of the experimental FTIR data established that catechin-loaded clinoptilolite matrices predominantly exhibited the characteristic peaks of the polyphenol, and the quantity of the encapsulated bioactive organic molecules increased with increasing catechin initial concentration.

### 2.4. Catechin Encapsulation Study

The effect of catechin initial concentration on the extent of its encapsulation by the mineral microparticles was investigated at equilibrium conditions with series of solutions with different polyphenol concentrations in the range 50–200 µg/cm^3^. The experimental data is presented in in Supplementary Materials Table S2. The maximum encapsulation capacity (EC) of CLS-5 (EC = 0.98 µg/mg) was attained at 200 µg/cm^3^ initial polyphenol concentration after 2 h, whilst the highest encapsulation efficiency (EE = 32%) was achieved at 50 µg/cm^3^ initial catechin concentration.

The mechanism of the processes of biologically active molecules’ incorporation into mineral networks via sorption is essential when designing novel drug-carrier systems as it reveals the rate-limiting stage/s, as well as the possible host–guest interactions between the physiologically active compounds and the recipients, which in turn provide a fundamental basis for explaining the in vitro release performance of the system. Mathematical modelling of mass transfer processes is the major tool to reveal this mechanism and to predict the behaviour of the system at varying operational conditions. Thus, nine mathematical models were applied to describe the process of catechin encapsulation into CLS-5 microparticles in the present study.

Some of the models generally consider the nature of the solid surface: homo- or hetero-geneous, and assist the distinguishment between mono- and multi-layer sorption, while others depict intermolecular interactions, pores/active sites distribution, process feasibility, etc. (Supplementary Material Table S3).

The values of the model parameters, regression coefficients (R^2^) and error functions: sum of squared errors (SSE), mean squared error (MSE), root-mean-square error (RMSE), of the Langmuir, Freundlich, Flory-Huggins, Fowler-Guggenheim, Temkin and the multilayer isotherm models (Table 1) were obtained through non-linear regression analyses.

**Figure 4:**
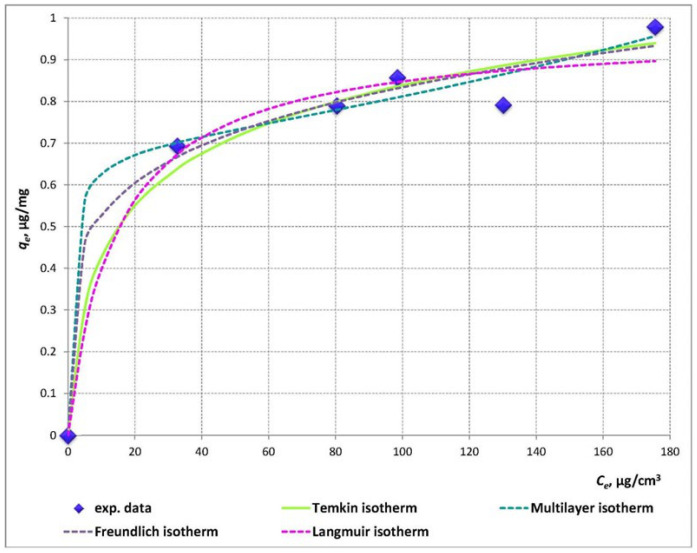
Experimental and model isotherms of catechin microencapsulation into clinosorbent-5 (CLS-5) framework.

According to Langmuir, adsorption is proportional to the fraction of the surface of the adsorbent that is open, while desorption is proportional to the fraction of the adsorbent surface that is covered. The Langmuir model assumes that the maximum sorption capacity corresponds to complete monolayer coverage of the molecules on the solid surface with no interaction between them. In addition, this empirical model refers to the same activation energy of sorption when the sorption of each molecule occurs at definite localised sites onto a homogeneous surface without transmigration of the adsorbate in the plane of the surface. The encapsulation monolayer capacity of clinoptilolite predicted by the Langmuir model (*q_m_*) (Table 1) was approximately equal to the experimentally obtained maximum capacity, i.e., according to the model assumptions, all catechin molecules are incorporated in the form of a monolayer on the solid particles surface. The latter concept was totally rejected by the assumptions and the proven significantly higher suitability of the multilayer model. Undoubtedly, according to the statistical and error analyses (Table 1) and to the mode of the model isotherm (Figure 4), the multilayer model suggests the best correlation with the experimental equilibrium data. The approximately equal values of the equilibrium constants for first layer (*K*_1_) and multilayer (*K*_2_) sorption suggest a comparable extent of catechin molecules’ monolayer formation and further multilayer encapsulation within the solid carrier pores and channels. Besides, the computed maximum monolayer capacity, *Q_m_* = 0.687 µg/mg, was 30% lower than the maximum experimentally determined capacity, which proves the presence of available vacant active sites within the solid particles, which could explain the formation of multilayers of catechin molecules.

Despite its lower applicability, the Fowler-Guggenheim model is essential for defining the mechanism of the encapsulation process, as the value of the parameter *W* is indicative for the nature of the interactions between the encapsulated molecules. A negative *W* value is associated with a decrease in the sorption heat during the encapsulation process, and with the existence of repulsive forces between the sorbed molecules. A value of *W* = 0 designates a lack of intermolecular interactions, while *W* > 0 denotes the presence of attractive forces between the organic molecules and increasing of the sorption heat throughout the encapsulation process. The high positive value of the latter parameter in the present study (*W* = 8.96) undoubtedly proves the presence of significant intermolecular attractive forces between catechin molecules.

The degree of surface coverage *θ*, according to the Flory-Huggins model, calculated as 1 − *C_e_/C_o_*, varies from 0.67 to 0.35 within the polyphenol concentration range 50–200 µg/cm^3^. Besides, the model predicts that the number of catechin molecules occupying sorption sites is *n_FH_* ≈ 0.25, which proposes availability of a significant number of vacant active sites. The latter serves as an indication of the absence of crowded conditions due to geometric and/or steric effects.

The effects of indirect sorbate/sorbate interactions during the encapsulation process are considered by the Temkin isotherm model. It also assumes linear decrease of sorption heat in the layer of sorbed organic molecules as a result of increased surface coverage [35,36,37,38,39,40]. The reasonably high value of the correlation coefficient and the low values of the SSE, MSE, and RMSE error functions suggest a satisfactory extent of correlation between the experimental and model data, which in turn presumes uniform distribution of the binding energies of catechin molecules on/within the solid CLS-5 microparticles, especially in the middle and high concentration range (Figure 4).

Considering the theoretical bases of the Freundlich model, the studied separation process could be described either as non-ideal and reversible sorption unrestricted to monolayer formation on a heterogeneous surface, or as multilayer sorption with non-uniform distribution of sorption heat and affinities over the heterogeneous surface [31]. Besides, the low value of the parameter *𝑛_F_* < 1 is indicative of a chemisorption process.

### 2.5. Thermodynamics Analyses

A frequent approach applied for the assessment of the compatibility of a drug in a microcarrier is the computation of the probable thermodynamic interactions. The thermodynamic parameters of catechin encapsulation into clinoptilolite particles were calculated on the basis of the applied equilibrium models. Generally, values of the Gibbs free energy, Δ*G^o^*, up to −20 kJ/mol are consistent with electrostatic interactions between the charged molecules and the charged sorbent surface (physisorption). Values of Δ*G^o^* ≈ −40 kJ/mol or higher are associated with chemisorption due to sharing or transfer of electrons from the bioflavonoid molecules to the clinoptilolite surface, leading to the formation of a coordinate type of bonding (chemisorption). In the present study, Δ*G^o^* was calculated on the basis of the values of the equilibrium parameters determined by each of the proposed models (Table 1). The values of Δ*G^o^* determined by the applied models are negative and higher than −20 kJ/mol, indicating that the encapsulation of catechin is a spontaneous process. The spontaneity ensures stability of the sorbed layer, which occurred probably due to physical sorption, i.e., electrostatic attractions prevail over chemisorption [41].

### 2.6. In Vitro Release Kinetics

Layered zeolites exhibit adjustable interlayer porosity which can be exploited for controlled drug delivery, allowing detailed investigation of the drug release because the structure of the carrier is known [15].

The quantitative estimation of biologically active substances’ release from carrier systems is significant for the assessment of their applicability and efficiency for biomedical and pharmaceutical purposes.

The experimental data of catechin in vitro release from CLS-5 microparticles is presented in Supplementary Materials Table S4. The release experiments were performed for a period of 24 h. The extended release period was determined by three major objectives: (i) to investigate the overall in vitro release profile of the bioflavonoid-carrier system, (ii) to examine the behaviour and identify any probable deviations in the physicochemical characteristics of the recipient and the biomolecules in an acidic pH, and (iii) to use the in vitro release experimental data for future studies on the antiproliferative activity of the designed catechin-CLS-5 micro-formulations on cancer cell lines with a cell cycle of approximately 24 h.

A variety of mathematical models have been developed and applied to correlate the experimental data and to give insights on the release phenomena [42]. In the present study, the kinetics of catechin in vitro release from catechin-loaded clinoptilolite microparticles was described by six mathematical models: the zero-order, first-order, Higuchi, Korsmeyer-Peppas, Weibull, and the sigmoidal function models.

The best-fitted model/s towards the experimental release data was/were evaluated by the values of the coefficients of determination (*R^2^*) and the error functions, as well as by the comparison between the experimental and model kinetics release curves. The model parameters and error functions are presented in Table 2.

The zero-order model describes drug release from a system that liberates its content with a constant rate, regardless of the concentration, and the release is only a function of time. The first-order model refers to a system for which the drug release rate is only a function of the remaining drug concentration. The Higuchi model implies that the amount of drug released form the dosage form is a function of the square root of time.

The Higuchi model assumes that the drug release occurs predominantly through Fickian diffusion and has typically been observed in drug-carrier systems with hydrophobic nature of the constituents. Higuchi applied Fick’s first law to describe the release of drugs in a boundary layer, from the surface of a pharmaceutical matrix towards an external solvent, which acts as a perfect sink under pseudo steady-state conditions. Since the assumptions of the model are approved only in the first part of the release process, the application of this law is recommended only for the first 60% of the release kinetics curve, i.e., *C_t_/C*_0_ = 0.6.

**Figure 5:**
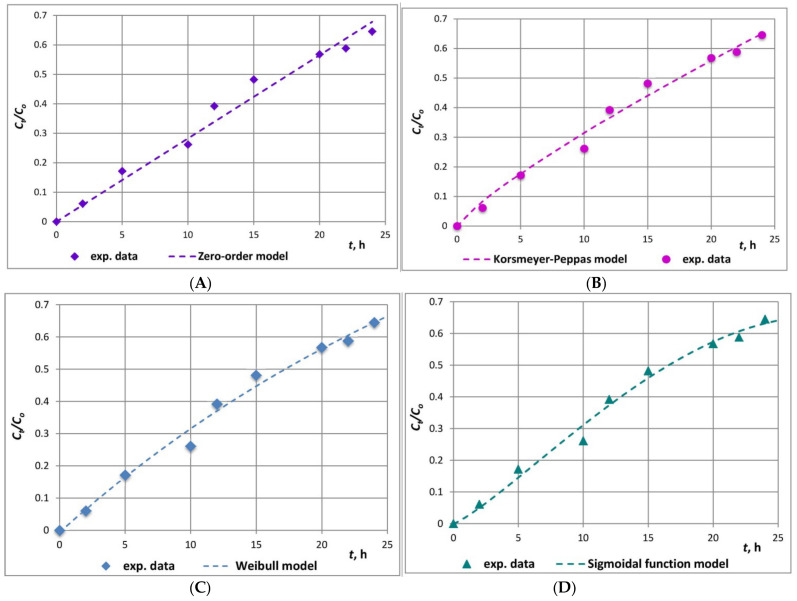
Experimental and (**A**) zero-order, (**B**) Korsmeyer-Peppas, (**C**) Weibull (*n* = 1), (**D**) sigmoidal function model in vitro release kinetics curves of catechin.

In the Korsmeyer-Peppas equation, the *n* value indicates the mechanism/s that describe/s release of an active compound from the carrier matrix [43]. Values of 0.45 ≤ *n* propose Fickian diffusion mechanism, 0.45 < *n* < 0.89—non-Fickian transport, *n* = 0.89—case II transport, and *n* > 0.89—super case II transport. The value of *n* = 0.83 computed in the present study indicates a probable alteration of catechin release mechanism from pseudo-Fickian diffusion to anomalous or non-Fickian release (Figure 5). However, as diffusion and relaxation effects are inherent for non-Fickian diffusion, greater number of parameters are necessary to consider the impact of both interacting mechanisms. Thus, the necessity of a proper multi-parameter mathematical model for the description of such complex release behaviour is imposed.

In this respect, the highest value of R^2^, the lowest SSE, MSE, and RMSE values (Table 2), and the mode of the model release curve (Figure 5) illustrate the overall superiority of the sigmoidal function model. The significantly higher value of the kinetics rate constant *k_s2_* as compared to *k_s1_* (Table 2) could be explained from the viewpoint of the existence of attractive intermolecular catechin–catechin interactions, which probably provoke slowing down of the release from initially highly loaded clinoptilolite active sites, followed by increased release rate at later times when the microcarrier sorption sites are no longer highly loaded. The latter inflicts sigmoidal release behaviour of the studied system. The inflexion point registered at approximately 35% release outlines an initial stage of low desorption rate and a second region, which is characteristic of increased release rate. The study and analysis of Loew et al. on the release kinetics of poorly water-soluble drug molecules from liposomal nanocarriers suggested that formation of aggregates between organic molecules could evoke sigmoidal behaviour during in vitro release [28].

The Weibull model is an empirical model which describes both immediate and delayed drug release from various delivery systems, which includes parameters that are sensitive to the different concentration ranges of the kinetics release profile. It could be regarded as a favourable one among the studied models. The parameter *b* determines the shape of the release curve and could be associated with physiological effects. Besides, the exponent *b* has been recently accepted as an indicator of the drug transport mechanism through the carrier matrix. Values of *b* ≤ 0.75 predict Fickian diffusion in fractal or Euclidian spaces: 0.75 < *b* < 1 is attributed to a mechanism combining Fick’s diffusion and swelling controlled transport, while *b* > 1 is associated with a complex release mechanism. The experimental data in the present study were best correlated in the case of the boundary value *b* = 1 (Table 2, Figure 5). The latter is indicative of exponential release profile and a complicated release mechanism of catechin molecules encompassing: Fickian diffusion, swelling controlled mass transfer, and non-Fickian (anomalous) mass transfer. On one hand, this implies that the Weibull equation is a flexible model with great potential to be fitted to various release patterns. On the other hand, the exponential parameter *b* could be utilised for prediction and/or optimisation of the release conditions of a drug-delivery system, as well as for the achievement of a desirable release profile in vivo [44].

Consequently, the comparative analyses of the observations, derived from the models with the highest extent of correlation: the sigmoidal law and the Weibull model, to the experimental data of catechin in vitro release from the clinoptilolite matrix, lead to mutually consistent conclusions.

**Figure 6:**
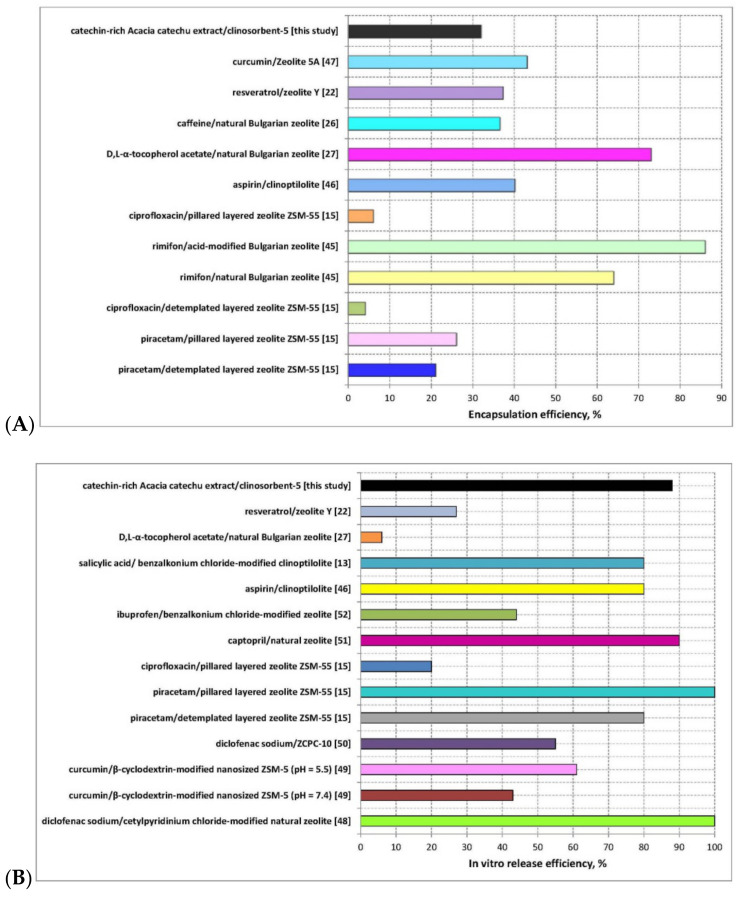
Literature review on the (**A**) encapsulation and (**B**) in vitro release efficiency of synthetic/natural bioactive substances-zeolite carrier systems [13,15,22,26,27,45,46,47,48,49,50,51,52].

Recent scientific studies on the design, applicability, encapsulation (Figure 6A), and in vitro release efficiency of innovative bioactive compounds-zeolite carrier systems (Figure 6B), outlined the competitiveness and reliability of the newly developed bioflavanol-CLS-5 micro-formulation in the present study. Significant comparability between the values of the cited encapsulation and release efficiencies and those obtained in the current investigations is observed.

### 2.7. Catechin–Clinoptilolite Interactions

**Figure 7:**
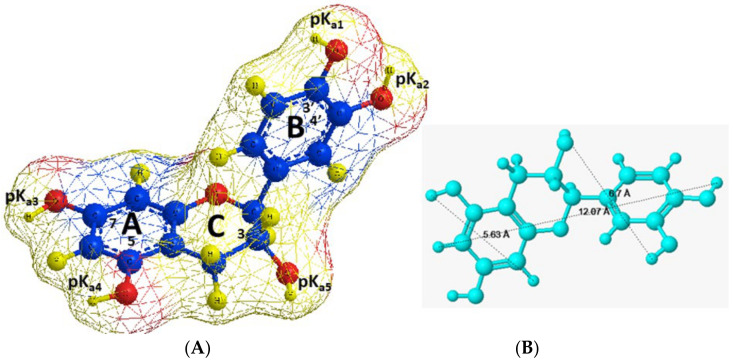
Catechin molecular structure, physicochemical, and molecular characteristics: (**A**) *pK_a_* values: *pK_a1_* ≈ 9.5, *pK_a2_* ≈ 8.9, *pK_a3_* ≈ 8.2–8.6, *pK_a4_* ≈ 10.3–11.2, *pK_a5_* ≈ 13.2–14.8 [53,54]. Connolly molecular area = 211.481 Å^2^. (**B**) Molecular dimensions [55,56].

Figure 7 presents the three-dimensional (3D) molecular structure, molecular dimensions, Connolly molecular area, and acid dissociation constants characteristic of the five OH-groups of the catechin molecule.

At pH values below the *pK_a_* values of catechin, the molecule undergoes protonation, while at higher pH values, it is in its neutral form. Therefore, possible electrostatic interactions between the A and B rings of the catechin molecules and the O-atoms of the clinoptilolite framework will be favoured at pH < *pK_a_* when the molecule is positively charged. The protonated drug could also participate in complex formation interactions with the Na^+^ cations present in the pore mouths of the alkalised mineral.

The clinoptilolite matrix is generally composed of two types of parallel channels (a, b), which are interconnected to a third type (c). The mouth of the channels has nominal dimensions of: *a* = 7.5 × 3.1 Å, *b* = 4.6 × 3.6 Å, and *c* = 4.7 × 2.8 Å [57]. However, their real dimensions are probably smaller than those mentioned above, due to the presence of extra-framework cations.

Therefore, the comparison of the dimensions of catechin molecules with those of the clinoptilolite channels revealed that the bioflavanol molecules are not spatially restricted to enter the clinoptilolite channels during the process of encapsulation. Thus, obviously, the interactions to be evaluated do not only concern the outer surface of the carrier. The incorporation of the bioflavanol molecules at pH = 4, which is lower than the *pK_a_* values, is probably facilitated by electrostatic attraction forces between the protonated positively charged organic molecules and the O-atoms within the carrier framework. Moreover, formation of coordinate bonding between the free electron orbital of the H^+^, serving as acceptors, and the unshared electron pair donated by the O-atoms, is also possible. These considerations based on the molecular characteristics and structural properties of the guest molecules and the recipient are consistent with the conclusions derived on the basis of the experimental studies and interpretative modelling.

However, possible obstacles and limitations during catechin encapsulation and release could arise from the possibility of intramolecular H-bonding between the H and O atoms of the neighbouring OH-groups in ring B, as well as from intermolecular H-bonding leading to the formation of catechin molecule aggregates [58].

## 3. Materials and Methods

### 3.1. Materials

The spray-dried extracts from *Acacia catechu* in powdered form originated from Northern India. (+)-catechin hydrate (C_15_H_14_O_6_xH_2_O) (≥98.0%, high-performance liquid chromatography (HPLC), powder) (CAS No.: 225937-10-0), epigallocatechin (C_15_H_14_O_7_, CAS No.: 970-74-1, analytical standard), C_2_H_5_OH (≥99.8%, HPLC), CH_3_COOH, HCl, NaOH (pure for analysis, HPLC) were supplied by Sigma (Sigma-Aldrich Chemie GmbH, Merck KGaA, Darmstadt, Germany). The alkalised clinoptilolite was supplied by Prof. Nikolai Popov, Mineralagro-Z Ltd., Sofia, Bulgaria. The mean particle size, determined by digital microscopy (Biomed Digital Binocular Microscope 40–1000×, China), was 30 µm.

The mineral product Clinosorbent-5 (CLS-5) was prepared by a methodology developed in 1992 in the Institute of Cryobiology and Food Technologies (ICFT) by a joint team of research associates from the ICFT, the Military Medical Academy, and the Institute of Applied Mineralogy at the Bulgarian Academy of Sciences. The product is an ecologically pure natural mineral sorbent (clinoptilolite from the Bulgarian deposits Beli Bair and Beli Plast in ratio 1:2) with significant sorption and ion exchange potential [59,60].

### 3.2. Surface Chemistry Characterisation of Alkalised Clinoptilolite

Surface chemistry of clinoptilolite was characterised by SEM and FTIR analyses. The FTIR spectra of catechin, alkalised clinoptilolite, and bioflavonoid-loaded microparticles were obtained with the potassium bromide (KBr) disc technique in the range 400–4000 cm^−1^ using a TENSOR 37 Bruker FTIR spectrometer (Bruker Optik GmbH, Ettlingen, Germany). A scanning electron microscope (SEM), Philips SEM 515 (Philips, Amsterdam, Netherlands), was used to obtain information about the surface topography of CLS-5.

### 3.3. UV-Vis Spectrophotometry

The concentration of catechin in the *Acacia catechu* 70% ethanolic extracts was determined by an adapted UV-Vis spectrophotometric methodology [23] using a DR 5000 UV-Vis Spectrophotometer (Hach Lange, D-40549 Düsseldorf, Germany), supplied with 10 mm quartz cuvette cells. All spectra were recorded in the UV region at *λ* = 280 nm at pH = 4, with 2 nm slit width, 900 nm·min^−1^ scan speed, and very high smoothing.

### 3.4. Microencapsulation Studies

Microencapsulation experiments were carried out by agitating predetermined amounts of clinoptilolite with 20 cm^3^ flavonoid ethanolic solutions, with initial concentrations in the range 50–200 μg/cm^3^ at temperature, T, 20 ± 2 °C. The extract/microcarrier systems were agitated on an IKA^®^KS 130 Basic Shaker at 180 rpm. Equilibrium was established after 24 h. Then, the flavonoid solutions were separated from the solid phase by centrifugation with Heraeus Labofuge 200 (Thermo Fisher Scientific, Waltham, MA USA) at 5300× *g* for 30 min and filtered using 0.45 µm membrane filters (LCW 916, Hach Lange, D-40549 Düsseldorf, Germany) to ensure the solutions were free from clinoptilolite particles before measuring the residual catechin concentration. The encapsulation capacity (EC, µg/mg) and efficiency (EE,%) of the alkalised clinoptilolite towards the studied flavonoid were calculated by Equations (1) [27] and (2) [32]:

(1) $$\mathrm{EC}=\frac{\left(C_{0}-C_{e}\right)\cdot V}{m}$$

(2) $$\mathrm{EE}=\;\frac{\mathrm{amount}\;\mathrm{of}\;\mathrm{released}\;\mathrm{bioflavonoid}}{\mathrm{total}\;\mathrm{amount}\;\mathrm{of}\;\mathrm{encapsulated}\;\mathrm{bioflavonoid}}\cdot 100$$

where *C*_0_ (μg/cm^3^) is the initial catechin concentration in the liquid phase, *C*_0_ (μg/cm^3^) is the equilibrium catechin concentration in the liquid phase, *m* (g) is the mass of clinoptilolite microparticles (g), and *V* is the liquid phase volume (cm^3^).

The effect of contact time and pH on the encapsulation behaviour and efficiency of the designed flavonoid-mineral carrier systems were investigated.

### 3.5. In Vitro Release Studies

The in vitro release kinetics experiments were conducted by agitating 0.37 g dried *Acacia catechu* extract-loaded clinoptilolite samples with 20 cm^3^ simulated enzyme-free gastric fluid solution (pH = 1.2) and temperature 37 ± 0.5 °C. The flavonoid/microcarrier systems were agitated on an IKA_KS 130 Basic Shaker at 180 rpm for 24 h. The concentration of released catechin in the liquid phase was determined spectrophotometrically at predetermined time intervals (2, 5, 10, 12, 15, 20, 22, and 24 h) by withdrawing 3.5 cm^3^ samples. Volume corrections when processing the experimental data were done by replacement of the drawn volume with simulated gastric fluid to avoid saturation of the remaining solution.

All experiments were carried out in triplicate, and the average values were taken to minimise random error. Blanks containing no biomolecules and replicates of each encapsulation/release point were used for each series of experiments.

### 3.6. Mathematical Modeling: Statistical and Error Function Analyses

The experimental encapsulation results were modelled by six equilibrium models: the two-parameter Langmuir, Freundlich, Flory-Huggins, and Fowler-Guggenheim models, and the three-parameter multilayer isotherm and Temkin models. The experimental release data was interpreted by six release mathematical models (zero-order, first-order, Higuchi, Korsmeyer-Peppas, Weibull, and the sigmoidal function model) through non-linear regression analyses.

The statistical and error function analyses were accomplished by XLSTAT statistical software for Excel.

## 4. Conclusions

The results from the detailed study on catechin-rich *Acacia catechu* extract encapsulation and in vitro release on/from CLS-5 microparticles approved a satisfactory extent (32%) of the bioflavanol encapsulation by the clinoptilolite micro-formulations in acidic medium. The investigations of the in vitro release kinetics in simulated gastric medium established significant release efficiency of 88%, achieved after 24 h. Besides, the comparative analyses of the bioactive substance in vitro release profile displayed that the newly designed catechin/CLS-5 microcarrier system provided sustained in vitro release behaviour without an initial burst effect. The interpretative modelling analyses of the observations, derived from the applied mathematical models with the highest extent of correlation: the sigmoidal law and the Weibull model, led to mutually consistent conclusions and provided valuable evidence for the practical use of these models in drug release phenomena from mineral micro-formulations. The results from the present study could serve as an essential platform for the design and development of innovative catechin-CLS-5 microcarrier systems for application in human and veterinary medicine.

## Figures and Tables

**Table 1:** Encapsulation mathematical models—model parameters and error function values.

Model	Model Parameters	Error Functions
Two-parameter models		
Langmuir	*q_m_* = 0.970 *K_L_* = 0.069 Δ*G^o^* = −6.735	R^2^ = 0.975 SSE = 0.015 MSE = 0.004 RMSE = 0.061
Freundlich	*K_F_* = 0.332 *n_F_* = 0.2 Δ*G^o^* = −2.778	**R^2^ = 0.982** SSE = 0.011 MSE = 0.002 RMSE = 0.047
Flory-Huggins Δ*G^o^* = *RT* *ln* (*K_FH_*)	*n_FH_* = 0.2478 *K_FH_* = 0.5294 Δ*G^o^* = −1.6022	R^2^ = 0.9668 SSE = 0.018 MSE = 0.006 RMSE = 0.063
Fowler-Guggenheim	*K_FG_* = 0.9535 *W* = 8.96 Δ*G^o^* = −0.120	R^2^ = 0.9258 SSE = 0.025 MSE = 0.011 RMSE = 0.075
Three-parameter models		
Multilayer isotherm	*Q_m_* = 0.687 *K_1_* = 0.844 *K_2_* = 0.842 Δ*G^o^* = −0.427	**R^2^ = 0.9893** SSE = 0.008 MSE = 0.003 RMSE = 0.053
Temkin	*b* = 14.0577 *K_1_* = 0.1792 *K*_2_ = 1.0788 Δ*G^o^* = −4.331	R^2^ = 0.977 SSE = 0.014 MSE = 0.004 RMSE = 0.060

In bold—the best fitting model.

**Table 2:** Kinetics models of catechin in vitro release model parameters and error functions values.

Kinetics Release Model	Model Parameters Values	Error Functions	Parameters Designation
**Zero-Order**	*k*_0_ = 0.692	R^2^ = 0.982 SSE = 5.863 MSE = 0.733 RMSE = 0.856	*k_o_*—zero-order rate constant
**First-Order**	*C*_0_ = 1.652 *k* = –0.108	R^2^ = 0.872 SSE = 0.202 MSE = 0.029 RMSE = 0.170	*k*—first-order rate constant *C*_0_—initial sorbate concentration, mg/mL
**Higuchi**	*k_H_* = 2.889	R^2^ = 0.978 SSE = 24.211 MSE = 3.459 RMSE = 1.860	*k_H_*—Higuchi rate constant
**Korsmeyer-Peppas**	at *C_t_/C*_0_ < 0.6 *n* = 0.83 *k_KP_* = 0.047	R^2^ = 0.987 SSE = 0.006 MSE = 0.001 RMSE = 0.028	*n*—Korsmeyer-Peppas exponent *k_KP_*—Korsmeyer-Peppas exponent
**Weibull**	*at b* = 0.2 *C_mod_* = 0.189 *a* = 0.466 *t* = 1.639 *at b* = 1 *C_mod_* = 1.390 *a* = 0.026 *t* = 0.174	R^2^ = 0.981 SSE = 0.010 MSE = 0.002 RMSE = 0.041 **R^2^ = 0.989** **SSE** **= 0.005** **MSE = 0.001** **RMSE = 0.029**	*t*—a location parameter denoting the lag time before the onset of the drug release procedure *b*—a shape parameter: *β* > 1—sigmoidal form, *β* < 1—parabolic graph *a*—Weibull model parameter *C_mod_*—model concentration
**Sigmoidal Function Model**	*k_s1_* = −9.403 × 10^−5^ *k_s2_* = 2.199 × 10^−2^ *n_s1_* = 2.6 *n_s2_* = 1.2	**R^2^ = 0.990** **SSE = 0.006** **MSE = 0.001** **RMSE = 0.032**	*k_s_*_1_*, k_s_*_2_—rate constants in the sigmoidal release model *n_s_*_1_*, n_s2_*—sigmoidal model exponents

In bold—the best fitting model.

## Data Availability

Data of the compounds are available from the authors.

## Supplementary Materials

The following are available online. Table S1: FTIR absorption bands designation of catechin-rich spray-dried extract, clinoptilolite, and catechin-loaded clinoptilolite, Table S2: Experimental data of catechin encapsulation by CLS-5 at equilibrium conditions, Table S3: Model assumptions and parameter designations of the encapsulation mathematical models, Table S4: Experimental kinetics data of catechin in vitro release from catechin-loaded CLS-5 microparticles.

## Sample Availability

Samples of the compounds are available from the authors.

## References

[1] Hovhannisyan V., Dong C., Chen S., Hovhannisyan V. (2017). Photodynamic dye adsorption and release performance of natural zeolite. Sci. Rep..

[2] Simona M., Camelia T., Margeta K., Farkaš A. (2019). Zeolites Applications in Veterinary Medicine. Zeolites—New Challenges.

[3] Zarkovic N., Zarkovic K., Kraij M., Borovic S., Sabolovic S., Poliakblazi M., Cipak A., Pacelic K. (2003). Anticancer and antioxidative effects of micronized zeolite clinoptilolite. Anticancer Res..

[4] Sverko V., Sobočanec S., Balog T., Colić M., Marotti T. (2004). Natural micronised clinoptilolite and clinoptilolite mixtures with *Urtica dioica* L. extract as possible antioxidants. Food Technol. Biotechnol..

[5] Jung B.G., Toan N.T., Cho S.J., Ko J.H., Jung Y.K., Lee B.G. (2010). Dietary aluminosilicate supplement enhances immune activity in mice and reinforces clearance of porcine circovirus type 2 in experimentally infected pigs. Vet. Microbiol..

[6] Papaioannou D., Katsoulos P.D., Panousis N., Karatzias H. (2005). The role of natural and synthetic zeolites as feed additives on the prevention and/or the treatment of certain farm animal diseases: A review. Microporous Mesoporous Mater..

[7] Tiwari J. (2008). Zeolite as Natural Feed Additives to Reduce Environmental Impacts of Swine Manure. Master’s Thesis.

[8] Tao Y.F., Qui Y., Fang S.Y., Liu Z.Y., Wang Y., Zhu J.H. (2010). Trapping the lead ion in multi-component aqueous solutions by natural clinoptilolite. J. Hazard. Mater..

[9] Topashka-Ancheva M., Beltcheva M., Metcheva R., Rojas J.A.H., Rodriguez-De la Fuente A.O., Gerasimova T., Rodríguez-Flores L.E., Teodorova S.E. (2012). Modified natural clinoptilolite detoxifies small mammal’s organism loaded with lead II: Genetic, cell, and physiological effects. Biol. Trace Elem. Res..

[10] Farias T., Ruiz-Salvador A.R., Rivera A. (2003). Interaction studies between drugs and a purified natural clinoptilolite. Microporous Mesoporous Mater..

[11] Rahimi M., Mobedi H., Behnamghader A., Baygi A.N., Mivehchi H., Biazar E. (2012). Fat-soluble vitamins release based on clinoptilolite zeolite as an oral drug delivery system. Lett. Drug Des. Discov..

[12] Khojaewa V., Lopatin O., Zelenikhin P., Ilinskaya O. (2019). Zeolites as carriers of antitumor ribonuclease binase. Front. Pharmacol..

[13] Jevtic S., Grujic S., Hrenovic J., Rajic N. (2012). Surfactant-modified clinoptilolite as a salicylate carrier, salicylate kinetic release and its antibacterial activity. Microporous Mesoporous Mater..

[14] Kraljević Pavelić S., Simović Medica J., Gumbarević D., Filošević A., Pržulj N., Pavelić K. (2018). Critical Review on Zeolite Clinoptilolite Safety and Medical Applications in vivo. Front. Pharmacol..

[15] Strzempek W., Korzeniowska A., Kowalczyk A., Roth W.J., Gil B. (2020). Detemplated and Pillared 2-Dimensional Zeolite ZSM-55 with Ferrierite Layer Topology as a Carrier for Drugs. Molecules.

[16] Putra M.W.A., Kusumawati G.A.W. (2018). The use of clinoptilolites as carrier of metformin hydrochloride in drug delivery system: In vitro drug release study. Asian J. Pharm. Clin. Res..

[17] Souza J.M.S., Sainz-Díaz C.I., Viseras C., Pergher S.B.C. (2020). Adsorption capacity evaluation of zeolites as carrier of isoniazid. Microporous Mesoporous Mater..

[18] Mavrodinova V., Popova M., Yoncheva K., Mihályc J., Szegedi A. (2015). Solid-state encapsulation of Ag and sulfadiazine on zeolite Y carrier. J. Colloid Interface Sci..

[19] Yaneva Z., Ivanova D. (2020). Catechins within the biopolymer matrix—design concepts and bioactivity prospects. Antioxidants.

[20] Salleh N., Mahat M.M., Yahaya S.M., Rosmamuhamadani R. (2020). Synthesis and characterization of cross-linked zerumbone loaded zeolite Y-gelatin for oral controlled release. AIP Conf. Proc..

[21] Karimi M., Habibizadeh M., Rostamizadeh K., Khatamian M., Divband B. (2019). Preparation and characterization of nanocomposites based on different zeolite frameworks as carriers for anticancer drug: Zeolite Y versus ZSM-5. Polym. Bull..

[22] Popova M., Yoncheva K., Szegedi A., Kalvachev Y., Benbassat N., Mavrodinova V. (2014). Resveratrol loading on mesoporous silica and zeolite carriers by solid state method. Bulg. Chem. Commun..

[23] Tomečková V., Reháková M., Mojžišová G., Wadsten T., Zelenáková K., Komanický V. (2016). Spectral study of modified natural clinoptilolite with pharmacologically active escin and horse chestnut extract. Spectrosc. Lett..

[24] Yaneva Z., Georgieva N., Bekirska L., Lavrova S. (2018). Drug mass transfer mechanism, thermodynamics, and in vitro release kinetics of antioxidant-encapsulated zeolite microparticles as a drug carrier system. Chem. Biochem. Eng. Q. CABEQ.

[25] Derakhshankhah H., Jafari S., Sarvari S., Barzegari E., Moakedi F., Ghorbani M., Shiri Varnamkhasti B., Jaymand M., Izadi Z., Tayebi L. (2020). Biomedical applications of zeolitic nanoparticles, with an emphasis on medical interventions. Int. J. Nanomed..

[26] Yaneva Z.L., Staleva M.S., Georgieva N.N. (2015). Study on the host-guest interactions during caffeine encapsulation into zeolite. Eur. J. Chem..

[27] Yaneva Z., Georgieva N., Staleva M. (2016). Development of D,L-a-tocopherol acetate/zeolite carrier system: Equilibrium study. Mon. Chem. Chem. Mon..

[28] Loew S., Fahr A., May S. (2011). Modeling the release kinetics of poorly water-soluble drug molecules from liposomal nanocarriers. J. Drug Deliv..

[29] Popov N., Popova T., Denev I., Shekerdjiisky R., Balabansky L., Timev I., Dimitrova E. (2014). “Clinodetox”—Effective detoxicant of heavy metals and radionuclides. Proceedings of the 8th Meeting of Hasumi International Research Foundation—Bulgaria: “Together in Cancer Control: Immunology, Viruses and Natural Remedies”.

[30] Yaneva Z., Ivanova D., Beev G., Besheva K. (2020). Quantification of catechin in *Acacia catechu* extract by non-derivative, first derivative UV/Vis spectrophotometry and FT-IR spectroscopy. Bulg. Chem. Commun..

[31] Ahmad M., Mudgi P., Gani A., Hamed F., Masoodi F.A., Maqsood S. (2019). Nano-encapsulation of catechin in starch nanoparticles: Characterization, release behavior and bioactivity retention during simulated in-vitro digestion. Food Chem..

[32] Yaneva Z.L. (2019). Nonsteroidal anti-inflammatory drug solid-state microencapsulation on green activated carbon—Mass transfer and host-guest interactions. Chem. Biochem. Eng. Q..

[33] Chen Y.M., Tsao T.M., Liu C.C., Huang P.M., Wang M.K. (2010). Polymerization of catechin catalyzed by Mn-, Fe- and Al-oxides. Colloids Surf. B Biointerfaces.

[34] Favvas E.P., Tsanaktsidis C.G., Sapalidis A.A., Tzilantonis G.T., Papageorgiou S.K., Mitropoulos A.C. (2016). Clinoptilolite, a natural zeolite material: Structural characterization and performance evaluation on its dehydration properties of hydrocarbon-based fuels. Microporous Mesoporous Mater..

[35] Ayawei N., Ebelegi A.N., Wankas D. (2017). Modelling and interpretation of adsorption isotherms. J. Chem..

[36] Yaneva Z., Koumanova B., Georgieva N. (2013). Linear and non-linear regression methods for equilibrium modelling of p-nitrophenol biosorption by *Rhizopus Oryzae*—Comparison of error analysis criteria. J. Chem..

[37] Georgieva N., Yaneva Z., Nikolova N. (2017). Direct Red 28 adsorption on Amosil and *Avena sativa* L.: Mass Transfer and kinetics modelling on the solid/solution interface. J. Solut. Chem..

[38] Yaneva Z., Georgieva N. (2017). Physicochemical and morphological characterization of pharmaceutical nanocarriers and mathematical modeling of drug encapsulation/release mass transfer processes. Nanoscale Fabrication, Optimization, Scale-Up and Biological Aspects of Pharmaceutical Nanotechnology.

[39] Yaneva Z., Georgieva N. (2014). Study on the physical chemistry, equilibrium, and kinetic mechanism of Azure A biosorption by *Zea Mays* biomass. J. Dispers. Sci. Technol..

[40] Yaneva Z., Koumanova B., Meshko V. (2010). Dynamic studies of nitrophenols adsorption on perfil in a fixed-bed column: Application of single and two resistance model. Water Sci. Technol..

[41] Georgieva N., Yaneva Z., Dermendzhieva D. (2017). Sorption equilibrium, thermodynamics and pH-indicator properties of cresyl violet dye/bentonite composite system. Water Sci. Technol..

[42] Freire M., Alexandrino F., Marcelino H.R., Picciani P., Silva K., Genre J., Oliveira A.G., Egito E. (2017). Understanding drug release data through thermodynamic analysis. Materials.

[43] Permanadewi I., Kumoro A.C., Wardhani D.H., Aryanti N. (2019). Modelling of controlled drug release in gastrointestinal tract simulation. J. Phys. Conf. Ser..

[44] Jahromia L.P., Ghazali M., Ashrafi H., Azadi A. (2020). A comparison of models for the analysis of the kinetics of drug release from PLGA-based nanoparticles. Heliyon.

[45] Georgieva N., Yaneva Z. (2015). Comparative evaluation of natural and acid-modified layered mineral materials as rimifon-carriers using UV/VIS, FTIR, and equilibrium sorption study. Cogent Chem..

[46] Tondar M., Parsa M.J., Yousefpour Y., Sharifi A.M., Shetab-Boushehri S.V. (2014). Feasibility of clinoptilolite application as a microporous carrier for pH-controlled oral delivery of aspirin. Acta Chim. Slov..

[47] Abadeh Z.A., Saviano G., Ballirano P., Santonicola M.G. (2020). Curcumin-loaded zeolite as anticancer drug carrier: Effect of curcumin adsorption on zeolite structure. Pure Appl. Chem..

[48] De Gennaro B., Catalanotti L., Cappelletti P., Langella A., Mercurio M., Serri C., Biondi M., Mayol L. (2015). Surface modified natural zeolite as a carrier for sustained diclofenac release: A preliminary feasibility study. Colloids Surf. B Biointerfaces.

[49] Amani S., Garmarudi A.B., Rahmania N., Khanmohammadi M. (2019). The β-cyclodextrin-modified nanosized ZSM-5 zeolite as a carrier for curcumin. RSC Adv..

[50] Krajišnik D., Daković A., Malenović A., Djekić L., Kragović M., Dobričić V., Milić J. (2013). An investigation of diclofenac sodium release from cetylpyridinium chloride-modified natural zeolite as a pharmaceutical excipient. Microporous Mesoporous Mater..

[51] Ainurofiq A., Choiri S. (2014). Application of montmorillonite, zeolite and hydrotalcite nanocomposite clays-drug as drug carrier of sustained release tablet dosage form. Indones. J. Pharm..

[52] Krajišnik D., Daković A., Malenović A., Kragović M., Milić J. (2015). Ibuprofen sorption and release by modified natural zeolites as prospective drug carriers. Clay Miner..

[53] Labidi N.S., Guerguer L., Kacemi A. (2018). Theoretical evaluation of antioxidant activity of tea catechins. J. Mater. Environ. Sci..

[54] Muzolf-Panek M., Gliszczyn’ska-S’wigło A., Szymusiak H., Tyrakowska B. (2012). The influence of stereochemistry on the antioxidant properties of catechin epimers. Eur. Food Res. Technol..

[55] Barak P., Nater E.A. The Virtual Museum of Minerals and Molecules. 1997–201x. http://virtual-museum.soils.wisc.edu.

[56] Barak P., Nater E.A. (2005). The Virtual Museum of Minerals and Molecules: Molecular visualization in a virtual hands-on museum. J. Nat. Resour. Life Sci. Educ..

[57] Petrov O.E., Karamaneva T.A., Kirov G.N. (1984). Cation distribution in clinoptilolite structure: Natural samples. Comptes Rendus Acad. Bulg. Sci..

[58] Uivarosi V., Munteanu A., Justino G.C. (2017). Flavonoid Complexes as Promising Anticancer Metallodrugs. Flavonoids: From Biosynthesis to Human Health.

[59] Popov N., Popova T., Zlatev A., Stanchev P., Marinov V., Belokonski I. Ion-exchange and sorption properties of clinoptilolite from Bulgarian deposits in respect to fractional and chemical composition. Proceedings of the Scientific-Practical Conference on People Prevention During Disasters and Accidents.

[60] Raynov N., Popov N., Yanev Y., Petrova P., Popova T., Hristova V., Atanasova R., Zankarska R., Kirov G., Filizova L., Petrov O. (1997). Geological, mineralogical and technological characteristics of zeolitized (clinoptilolitized) tuffs deposits in the Eastern Rhodopes, Bulgaria. Natural Zeolites.

