# Supplementary material for: Clinoptilolite Microparticles as Carriers of Catechin-Rich Acacia catechu Extracts: Microencapsulation and In Vitro Release Study
Source: Molecules. 2021 Mar 16;26(6):1655. doi: 10.3390/molecules26061655 (PMC8002362; doi:10.3390/molecules26061655)
Supplement: Supplementary file 1 [file molecules-26-01655-s001.zip › molecules-1139964-supplementary.pdf]

molecules

MDPI

Article

Clinoptilolite Microparticles as Carriers of Catechin-Rich *Acacia Catechu* Extracts: Microencapsulation and In Vitro Release Study

Zvezdelina Yaneva [1,*], Donika Ivanova [1] and Nikolay Popov [2]

[1] Chemistry Unit, Department of Pharmacology, Animal Physiology and Physiological Chemistry, Faculty of Veterinary Medicine, Trakia University, Students Campus, 6000 Stara Zagora, Bulgaria
[2] Mineralagro-Z Ltd, Sofia, Bulgaria.
* Correspondence: z.yaneva@abv.bg; zvezdelinayaneva@trakia-uni.bg; Tel.: (+359 898 399 203)

Supplementary Table S1. FT-IR absorption bands designation of catechin-rich spray-dried extract, clinoptilolite and catechin-loaded clinoptilolite.

Wavenumber, cm^{-1}	Catechin	Clinoptilolite	Catechin-CLS-5 Microparticles
3600–3450	-	3654; 3450 lattice termination silanol groups located on the external surface, and bridging OH-groups with Bronsted acidity	3637**
3500–3400	3516; 3410 O–H linkage of phenolic groups; aromatic ring quadrant; intermolecular H-bonded phenolic –OH groups	-	3438*
2380–2340	2374; 2347 C-H-stretching vibrations in aromatic ring	-	2370; 2345*
1650–1630	1630 C=C aromatic ring stretching vibrations	bending vibration of water	1647–1645*
1300–1000	1282–1029 C–O stretching vibrations; C-O-H bending; plane bending vibrations of phenyl group; H-bonding	1060 Internal tetrahedral asymmetric stretching vibrations	1060*;**
880–840	879; 844 benzene ring vibrations	-	835*

700–600	620<br>CH deformation of aromatic rings;<br>615–600<br>C–C deformations; CH_2 -in-plane rocking;	O-H bending bands	615–600*
570–490	565; 503<br>C=C–C-aromatic ring asymmetric bending;<br>C–H-out-of-plane aromatic ring bending	-	565; 499*

*attributed to catechin

**attributed to clinoptlilolite

Supplementary Table S2. Experimental data of catechin encapsulation by CLS-5 at equilibrium conditions.

**Initial Catechin Concentration, C_o, μg/cm³**	**Equilibrium Catechin Concentration in the Liquid phase ± SD, C_e, μg/cm³**	**Encapsulation Capacity, *EC*, μg/mg**	**Encapsulation Efficiency, *EE*, %**
50	32.66 ± 0.15	0.69	34.68
100	80.25 ± 0.20	0.79	19.75
120	98.56 ± 0.17	0.86	17.87
150	130.20 ± 0.11	0.79	13.2
200	175.52 ± 0.19	0.98	12.24

Supplementary Table S3. Model assumptions and parameter designations of the encapsulation mathematical models.

Model	Model Assumptions	Parameter Designations
Langmuir	- accounts for the surface coverage by balancing the relative rates of adsorption and desorption (dynamic equilibrium).	K_L - Langmuir constant related to sorption capacity, $cm^3/\mu g$<br>q_m – monolayer capacity, μg/mg
Freundlich	- surface heterogeneity;<br>- defines exponential distribution of active sites and their energies;	K_F - sorption capacity ($cm^3/\mu g$)<br>$1/n_F$ – sorption intensity;
Multilayer isotherm	- multilayer encapsulation	K_1 - equilibrium constant for the first layer sorption;<br>K_2 - equilibrium constant for multilayer sorption;<br>Q_m - maximum monolayer sorption capacity, μg/mg
Fowler-Guggenheim	- lateral interactions between encapsulated molecules	K_{FG} - Fowler-Guggenheim equilibrium constant ($cm^3/\mu g$);<br>W - interaction energy between sorbed molecules (J/mmol);<br>R - the universal gas constant (R = 8.314 J/(mol·K);<br>T - absolute temperature (K).
Kiselev	- localized monomolecular layer sorption<br>- valid for surface coverage $q_e/q_{max} > 0.68$	K_i - Kiselev equilibrium constant (mL/μg)<br>Kn - constant of complex formation between encapsulated molecules
Harkin-Jura	-existence of heterogeneous pore distribution on the carrier surface;<br>- multilayer sorption;	A, B – Harkin-Jura constants
Temkin	- indirect interactions between the encapsulated molecules;<br>- the heat of sorption of all molecules decreases linearly with surface coverage;<br>- uniform distribution of the binding energies up to a definite maximum value.	b – constant related to sorption heat, J/mol<br>K_1, K_2 - Temkin isotherm constant
Halsey	- multilayer sorption at a relatively large distance from the surface<br>- heteroporous nature of the adsorbent	K_H, n - Halsey isotherm constants
Flory-Huggins<br>$\Delta G^o = RT \ln (K_{FH})$,	- assessment of the degree of surface coverage characteristics of the sorbate molecules on the carrier surface;<br>- expresses the feasibility and spontaneity of a sorption process	θ - degree of surface coverage;<br>n - number of sorbate molecules occupying active sites;<br>K_{FH} - Flory-Huggins equilibrium constant (mL/mmol);<br>ΔG^o - standard Gibbs free energy change

Supplementary Table S4. Experimental kinetics data of catechin *in vitro* release from catechin-loaded CLS-5 microparticles.

Time, t, h	Concentration of Released Catechin in the Liquid Phase ± SD, C_t, µg/cm³	Dimensionless Concentration of Released Catechin, C_t/C_o, -
2	1.5 ± 0.0643	0.061
5	4.2 ± 0.0950	0.172
10	6.4 ± 0.0635	0.261
12	9.6 ± 0.0793	0.392
15	11.8 ± 0.0695	0.482
20	13.9 ± 0.0764	0.568
22	14.4 ± 0.0878	0.588
24	15.8 ± 0.0737	0.645
